# Fibronectin-1 is a dominant mechanism for rheumatoid arthritis via the mediation of synovial fibroblasts activity

**DOI:** 10.3389/fcell.2022.1010114

**Published:** 2022-09-26

**Authors:** Jie Yang, Yan Zhang, Jingqi Liang, Xinquan Yang, Liang Liu, Hongmou Zhao

**Affiliations:** Department of Foot and Ankle Surgery, Honghui Hospital of Xi’an Jiaotong University, Xi’an , China

**Keywords:** rheumatoid arthritis, scrna-seq, fibronectin-1, synovial fibroblasts, cell function

## Abstract

Rheumatoid arthritis (RA) has a high incidence and adverse effects on patients, thus posing a serious threat to people’s life and health. However, the underlying mechanisms regarding the development of RA are still elusive. Herein, we aimed to evaluate the RA-associated molecular mechanisms using the scRNA-seq technique. We used the GEO database to obtain scRNA-seq datasets for synovial fibroblasts (SFs) from RA cases, and the genes were then analyzed using principal component analysis (PCA) and T-Stochastic Neighbor Embedding (TSNE) analyses. Bioinformatics evaluations were carried out for asserting the highly enriched signaling pathways linked to the marker genes, and the key genes related to RA initiation were further identified. According to the obtained results, 3 cell types (0, 1, and 2) were identified by TSNE and some marker genes were statistically upregulated in cell type 1 than the other cell types. These marker genes predominantly contributed to extracellular matrix (ECM) architecture, collagen-harboring ECM, and ECM structural components, and identified as enriched with PI3K/AKT signaling cascade. Notably, fibronectin-1 (FN-1) has been identified as a critical gene that is strongly linked to the development of SFs and has enormous promise for regulating the onset of RA. Moreover, such an investigation offers novel perspectives within onset/progression of RA, suggesting that FN-1 may be a key therapeutic target for RA therapies.

## Introduction

Rheumatoid Arthritis (RA) is a long-term, progressive autoimmune condition affecting more than 1% of the global population, inflicting severe pain, joint distortion, and disability (Newsome and American College of Rheumatology, 2002; Guo et al., 2018; van der Woude and van der Helm-van Mil, 2018). RA can affect not only the bones and joints, but also extra-articular organs (e.g., the heart, eyes, lungs, and blood vessels) and shorten the life expectancy (Conforti et al., 2021; Figus et al., 2021). Furthermore, there are six developing stages of RA i.e., pre-RA to RA stage, and various factors including genetic, environmental, and autoimmune factors can affect RA progression (Deane and Holers, 2021). Early treatment can alleviate joint malformation and joint damage. However, due to the unknown mechanisms of RA, present treatments for RA are mostly focused on symptom relief, malformation correction, and functional rehabilitation exercise, but the overall efficacy is far from satisfactory (Radu and Bungau, 2021). Hence, it is needed to explore RA-associated critical genes and uncover more useful treatment targets for RA.

The reported studies have revealed that synovial fibroblasts (SFs) considerably contribute to the development of RA (Tsuchiya et al., 2021). Joint inflammation is often accompanied by the proliferation of SFs, which leads to synovial thickening and even joint deformity (Müller-Ladner et al., 2007). Excessive proliferation of SFs can invade articular cartilage and surrounding tissues, while locally secreting a large number of inflammatory factors, such as IL-8, IL-1β, and other cytokines into local joints. The underlined process causes the inflammation to worsen (Ritchlin, 2000). Therefore, it is important to uncover the potential mechanisms of SFs formation as well as the key hub genes in this regulation process in order to have a thorough understanding of RA initiation and the crucial genes involved.

In recent decades, several RA-associated genes have been reported (Liu et al., 2020; Wang et al., 2020). Wang et al. reported that FoxC1 and β-catenin are closely related to the function of SFs(Wang et al., 2020). Furthermore, the *in vitro* and *in vivo* investigation showed that up-regulating FoxC1 could improve SFs’ proliferative, migratory, and invading abilities (Wang et al., 2020). Moreover, the pro-inflammatory cytokines were also markedly elevated in the FoxC1-treated SFs(Wang et al., 2020). Similarly, Liu et al. demonstrated the potential therapeutic impact of pyruvate dehydrogenase kinase 4 (PDK4) in inhibiting the development of RA (Liu et al., 2020). In addition, the rapid development of RNA sequencing technology has enabled the identification of a wide range of pivotal genes linked to disease onset (Hong et al., 2020). ScRNA-seq has been attracting accumulative interest in identifying the key genes in many diseases and be potentially employed as a major technique to evaluate mechanistics within RA initiation (Slovin et al., 2021; Grandi et al., 2022; Micheroli et al., 2022).

Hence, the current study aimed to evaluate the RA-associated molecular mechanisms using the scRNA-seq technique. Herein, scRNA-seq datasets for SFs from RA patients were retrieved from the GEO database, with genes undergoing principal component analysis (PCA) and T-stochastic Neighbor Embedding (TSNE) analyses. A bioinformatics analysis was conducted to determine the highly enriched signaling pathways linked to genomic biomarkers, with the essential gene associated with the initiation of RA was also identified.

## Methods

### Data searching and processing

This investigation probed GEO (Gene Expression Omnibus) (https://www.ncbi.nlm.nih.gov/geo/) and selected single-cell RNA-seq of SFs in patients with RA. For additional analysis, the gene count file was obtained. Three factors were chosen for data processing: nFeature (gene quantity/cell), nCount (total quantity of all genes/cell), and percent. mt (percentage of mitochondrial gene quantity within total gene quantity/cell). Cells having nFeature >50 and percent. mt < 5% were chosen for further study since mitochondrial gene expression was commonly found low.

### PCA and TSNE evaluations

Computational single-cell RNA-seq (scRNA-seq) methods have been successfully applied to experiments representing a single condition, technology, or species to discover and define cellular phenotypes. In Rstudio, R package “limma”, “Seurat”, and “dplyr” were employed to conduct data normalization, PCA, and TSNE analyses, in accordance with Andrew’s guidelines (Butler et al., 2018).

### Hub genes evaluation, protein-protein network development, and biological function analysis

DEGs evaluation, GO (Gene Ontology) and KEGG (Kyoto Encyclopedia of Genes and Genomes) analyses and visualization were carried out using the R packages “SingleR,” “org.Hs.eg.db,” “clusterProfiler,” “enrichplot,” and “ggplot2.” PPI network was constructed using Cytoscape 3.7.2 and STRING (http://string-db.org/).

## Results

### Data retrieval and quality assurance

**FIGURE 1:**
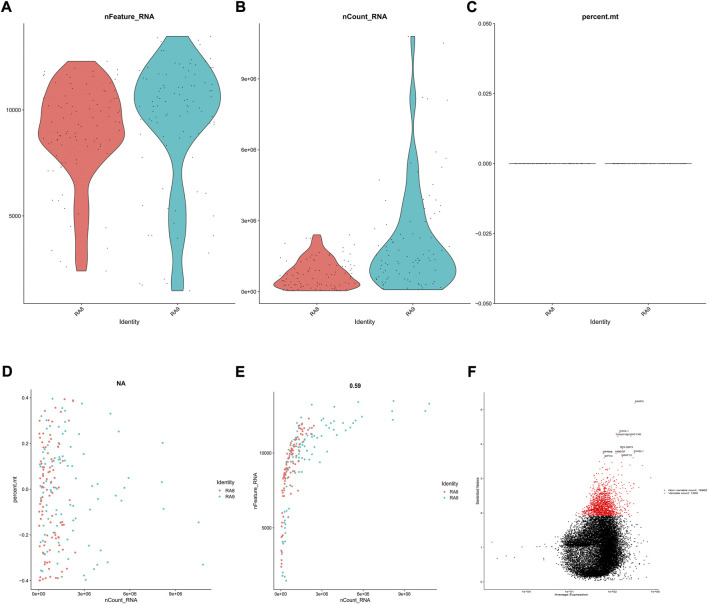
Evaluation of SFs’ scRNA-seq results. GSE109449 provided access to scRNA-seq datasets for 192 SFs obtained from two RA cases (RA8 and RA9), followed by evaluation in three aspects including **(A)** nFeature **(B)** nCount, and **(C)** percent. mt, as depicted within violin-plots. Next, correlation analyses were conducted on **(D)** nCount, percent. mt and **(E)** nCount and nFeature, and **(F)** Genes with greater expression changes in various cells were investigated using a scatter diagram. The vertical axis represents standardized variance, the abscissa represents average expression, and the red and black pots represent the top 1,000 and lower 1,000 genes, accordingly.

GSE109449 was obtained from the GEO database and was comprised of SFs transcriptomic data from two OA4 and two OA5 patients. A total of 192 cells were obtained with information about the number of genes in RA8 and RA9. nFeature was greater than 50 in each cell, whereas nCount and percent. mt were found to be typically less than 5,000,000 and 10%, accordingly, as depicted in Figures 1A–C. Post performing correlation analysis following nCount/percent. mt/nFeature, it was observed that there was no significant association between percent. mt and nCount. However, a significant correlation was recorded between nFeature and nCount (Figures 1D,E). Such dataset outcomes shed additional light upon downregulations within mitochondria. A scatter diagram was generated for identifying upregulated genes within various cell cultures (Figure 1F), followed by selecting the top 1,000 genes for the subsequent studies.

### PCA and TSNE evaluations

**FIGURE 2:**
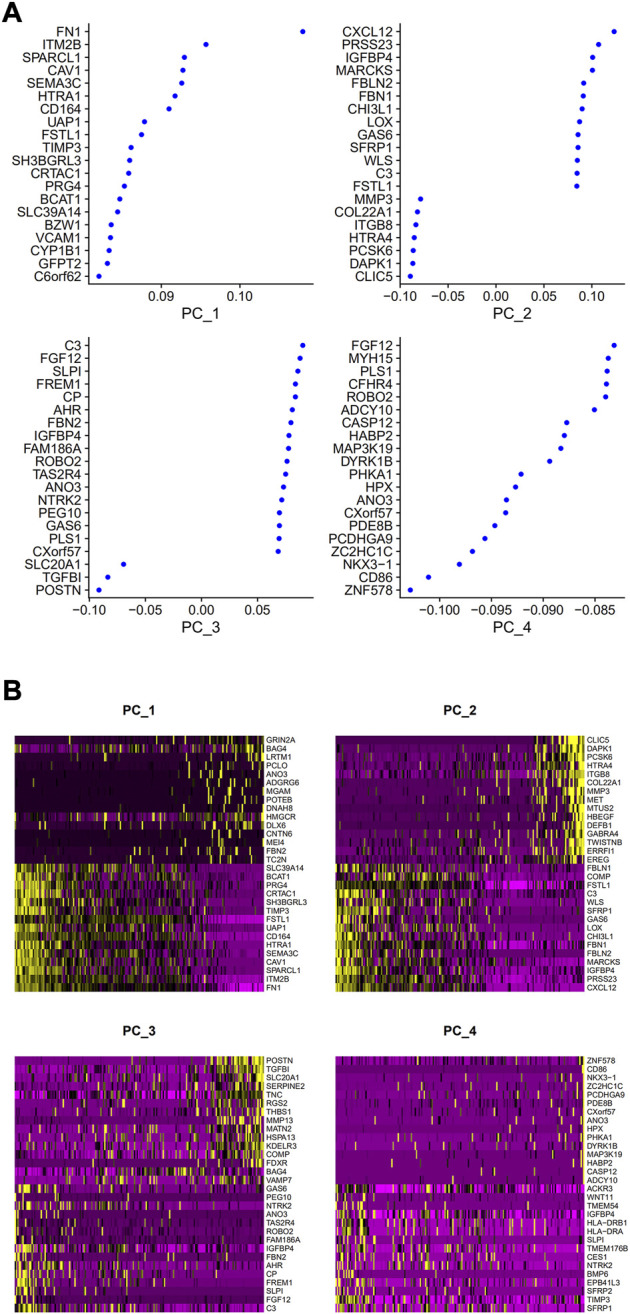
Top genes in PC1, 2, 3, 4. **(A)** Correlation coefficient data. **(B)** Data regarding gene expression.

**FIGURE 3:**
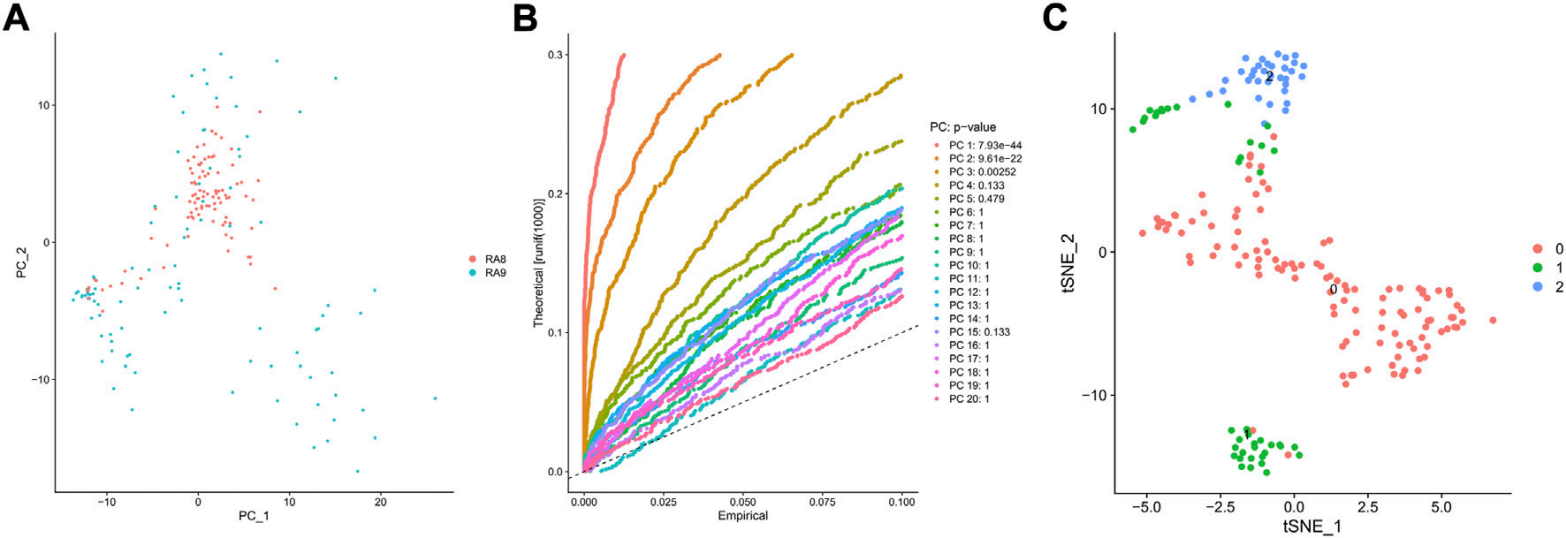
PCA and TSNE evaluations. **(A)** PCA was used to examine the 1,000 variant genes that had previously been screened, and 20 PCs were ultimately found. **(B)** The clustering of the 20 PCs was performed, and 3 PCs with the most considerable variations were evaluated. A theoretical value is represented by the vertical axis, whereas an empirical value is represented by the abscissa. The TSNE analysis was then used to successively cluster the **(C)** cells within such 3 PCs into three cell-types.

**FIGURE 4:**
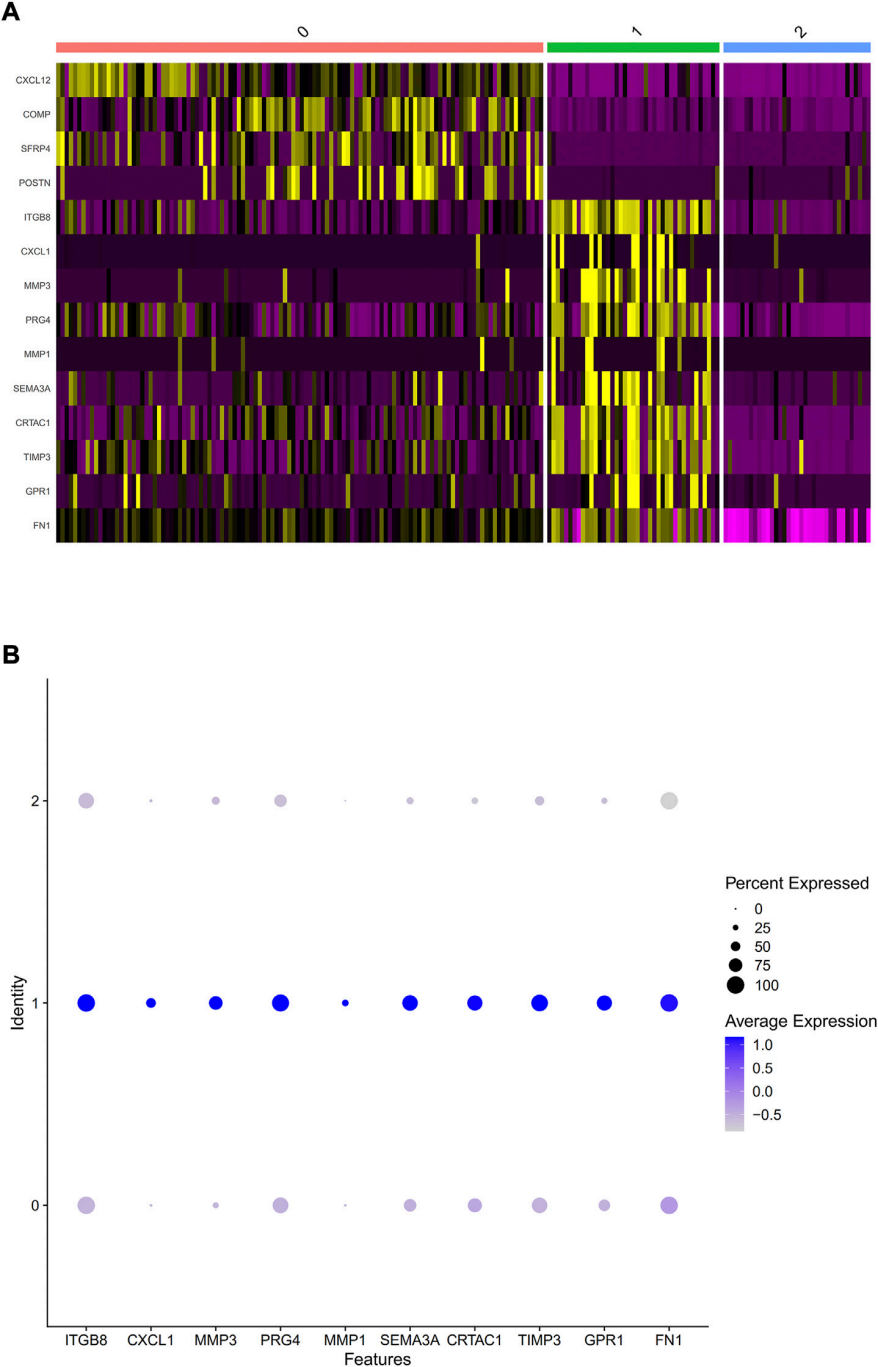
Selection of considerably divergent biomarker-genes within TSNE cell-types. **(A)** A heatmap was used to show differing gene dysregulations within three cell-types, whereby which purple and yellow color depicts downregulated and upregulated genes, accordingly. The abscissa indicates cells of types 0, 1, and 2, while vertical-axis displays normalized biomarkers for three cell-types employing Seurat 3.0. **(B)** The expression data for ten marker genes in type 1 cells.

**FIGURE 5:**
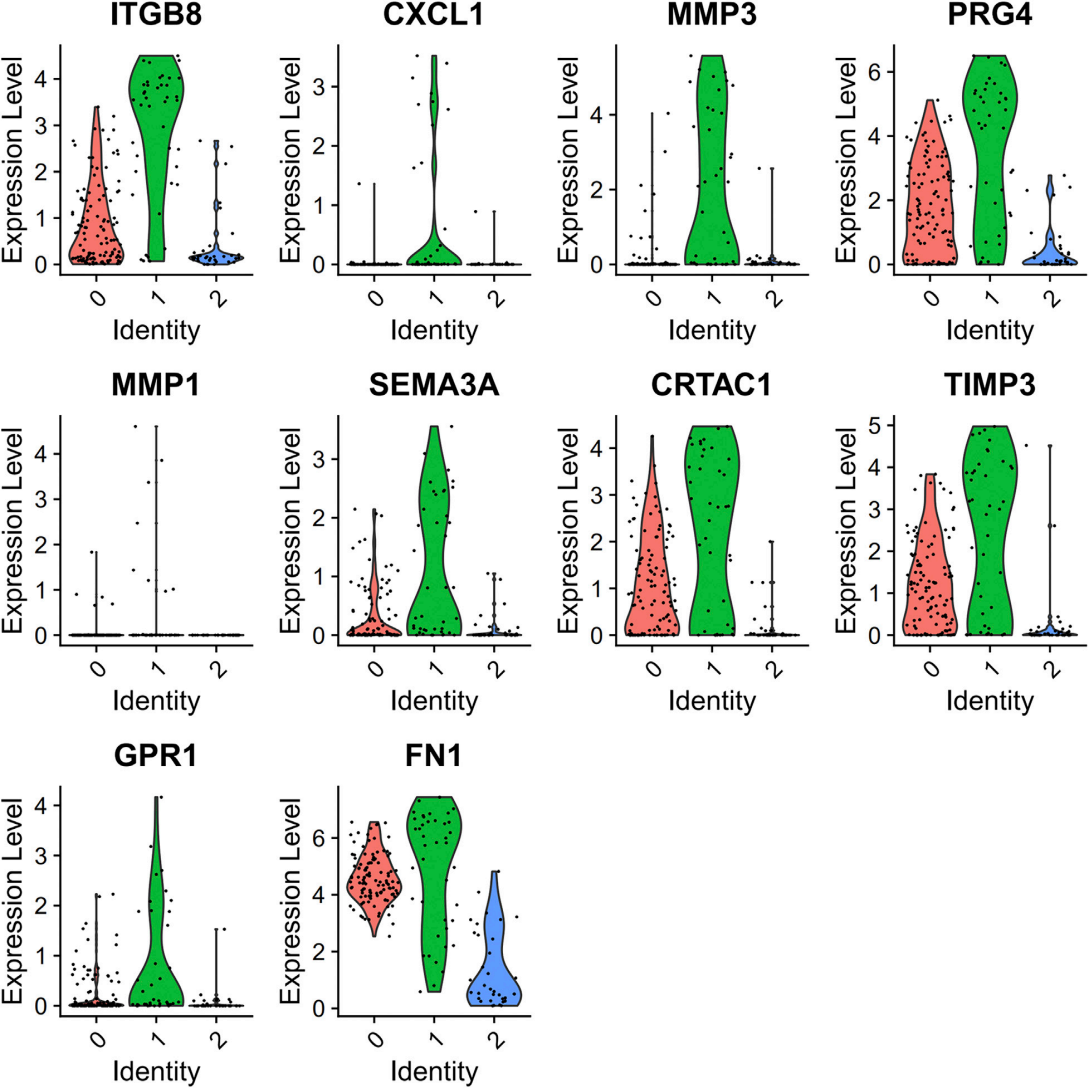
Data regarding 10 marker genes of type 1. Expression analysis within type-1 cultures, with red and green, indicates low- and high-expression, accordingly.

**FIGURE 6:**
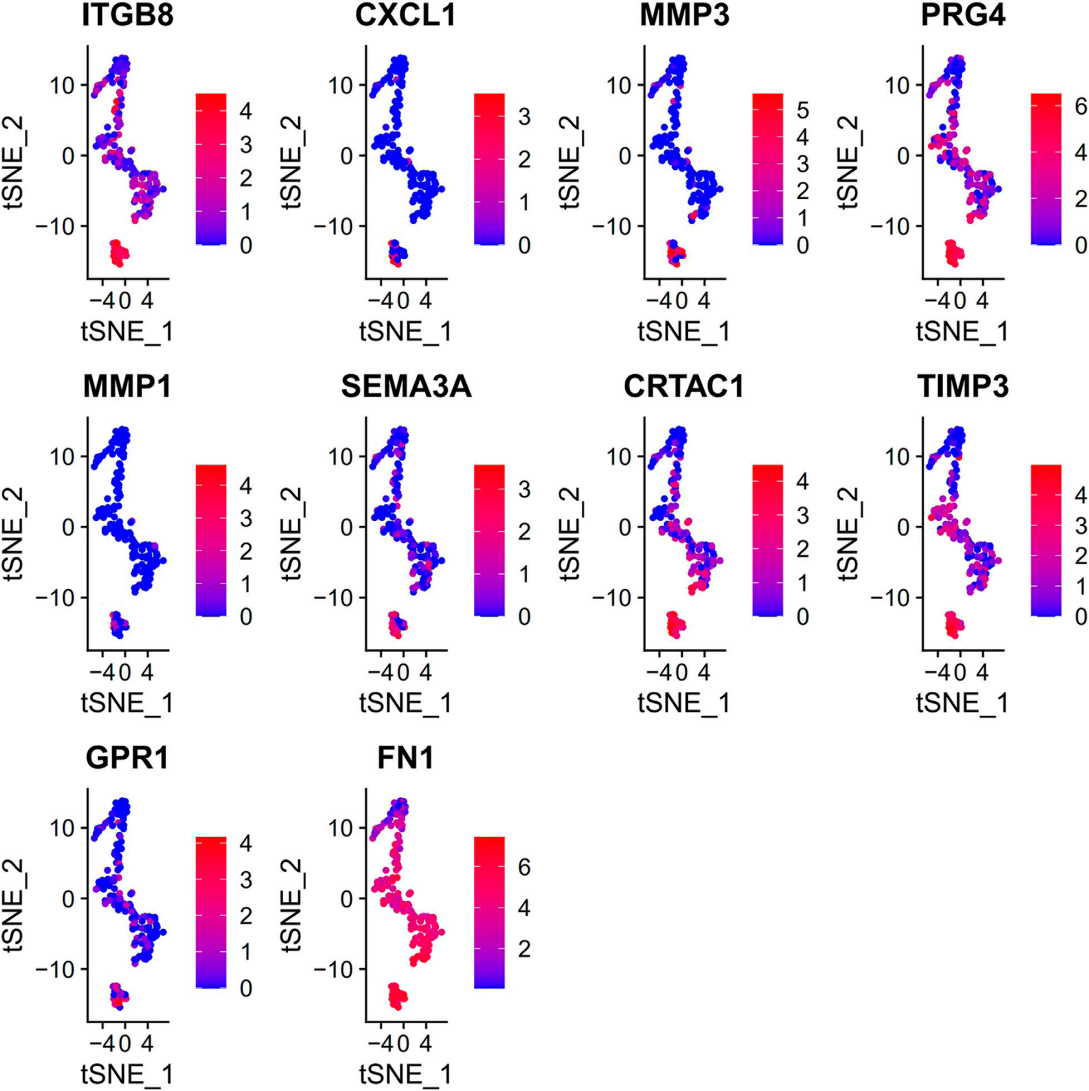
Information details of 10 marker genes of type 1. Evaluation of the underlined genes in three types of cells, with abscissa and the vertical axis indicating cell types and expression levels, accordingly.

PCA was employed to determine the biomarker genes from a set of the top 1,000 previously analyzed genes. In addition, 20 principal components (PCs) were found. The underlined top genes in PC1-4 are shown in Figure 2. Discreteness was clearly shown within cellular spread for the top-two PCs, i.e., PC1 and PC2. The results (Figure 3A) demonstrated that dimensionality reduction and cell classification could be accomplished by PCA evaluations. Next, the cells in the underlined 20 PCs were evaluated by cluster analysis, and ultimately, three PCs, i.e., PC1, PC2, and PC3 with obvious differences, were determined (Figure 3B). By employing the TSNE analysis, the cultures within such three PCs were subsequently divided into three cell-types (i.e., type-0, type-1, and type-2), as shown in Figure 3C. Next, the genes within each type were differentially examined to identify biomarker genes (log | FC | > 0.5, *p*-value < 0.05). Since all three cell-types have no considerable correlation with one another, follow-up enrichment analysis was limited to genomic biomarkers that were considerably divergent across all such cell-types. Peak 10 genomic biomarkers for each cell-type were chosen in accordance with the log|FC| value in order to more accurately determine the characteristic genes with notable expression patterns in cells. Expression of all 30 biomarker genes was normalized and visualized using Seurat 3.0 (https://satijalab.org/seurat/), and 14 marker genes with significantly different expressions were then evaluated (Figure 4A). Based on the statistical evaluation, 10 biomarker genes (including, ITGB8, CXCL1, MMP3, PRG4, MMP1, SEMA3A, CRTAC1, TIMP3, GPR1, and FN-1) in type-1 cells were considerably more abundant in comparison to type-2 cells (Figure 4B). Next, expression profiles for 10 biomarker genes within type-0, type-1, and type-2 cells were isolated for visualization analysis, which revealed that type-1 cells had marked upregulation for 10 genes in comparison to remaining cell-types (Figure 5 and Figure 6). In view of these results, genomic biomarkers within TSNE type-1 had markedly divergent expression in comparison to other gene types. Furthermore, among these genes, features related to SF growth were more abundantly present.

### GO and KEGG enrichment analysis

**FIGURE 7:**
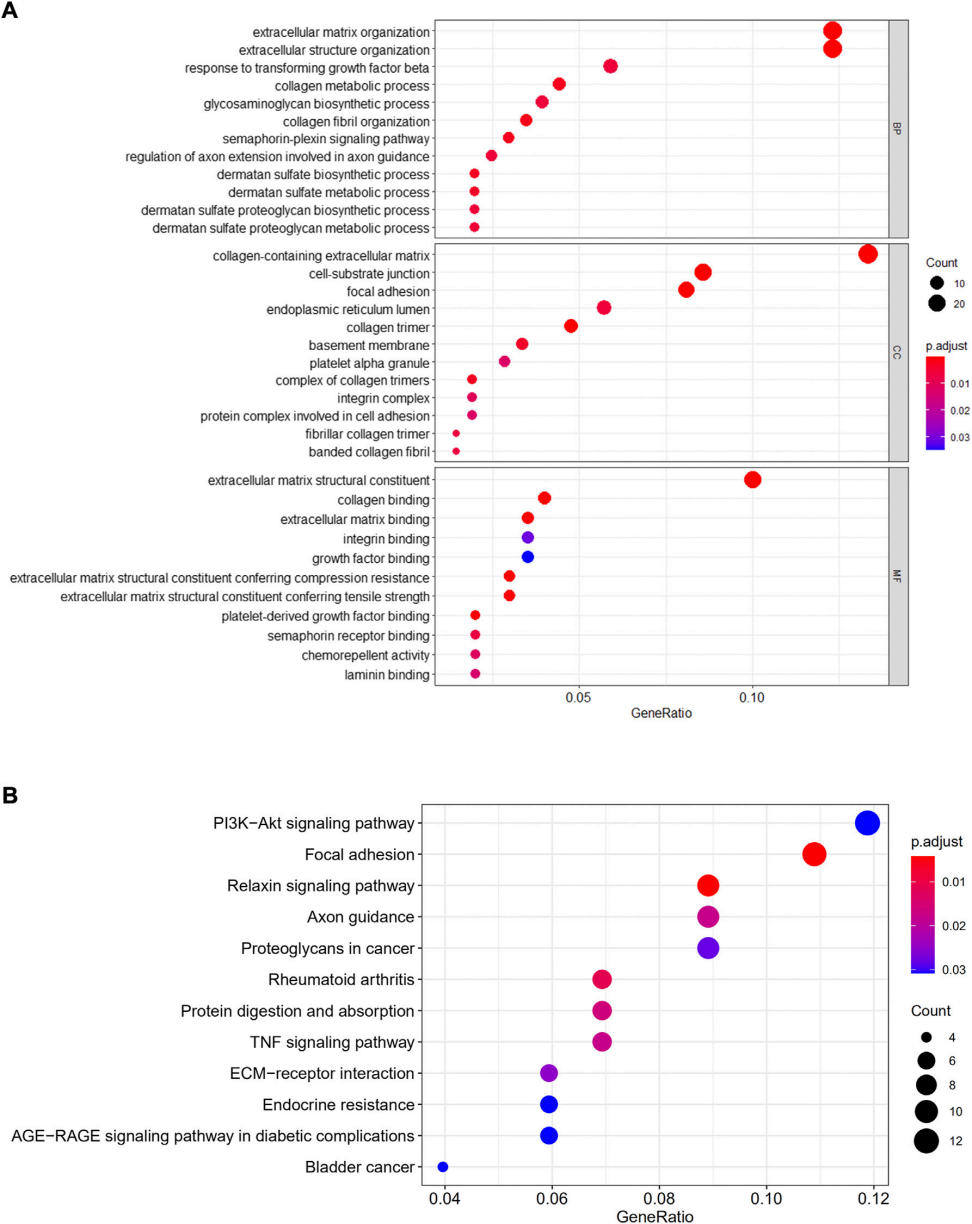
GO/KEGG enrichment analyses. GO **(A)** and KEGG **(B)** were performed for evaluating functional enrichments gene cascades within cell-type-1.

The cell type-1 biomarker genes were subjected to GO and KEGG analysis. The findings showed that genes in type-1 cells were primarily active in ECM architecture, and cell-adhesion molecular-binding (Figure 7A). In addition, they were enriched in PI3K/AKT signaling cascade, relaxin signaling cascade, axon guidance, and focal adhesion cascade, proteoglycans in the cascades associated with various carcinomas, AGE-RAGE signaling cascade in diabetic complications, RA cascade, Protein digestion, and absorption cascade, ECM-receptor interaction and TNF signal transduction cascades (Figure 7B).

### PPI network and evaluation of hub genes

**FIGURE 8:**
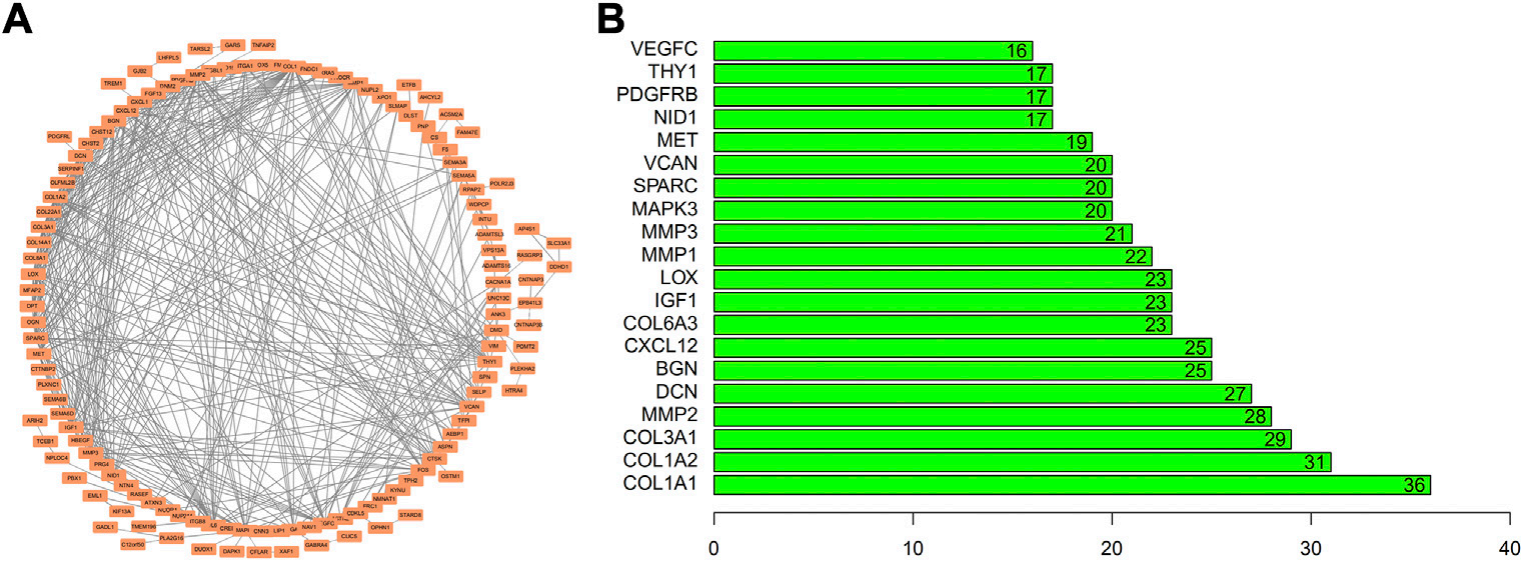
Development of PPI network/pivotal gene evaluations. Biomarkers for TSNE cell-type-1 were applied upon **(A)** PPI network and assessed **(B)** through quantifying nodes. Abscissa is displayed as the node quantity.

The STRING database (https://string-db.org/) was employed for map biomarker genes in cell type-1 to a PPI network. The underlined biomarker genes displayed a specific linkage with some genes in the network, indicating that they may influence SFs through reciprocal adjustment (Figure 8A). It was revealed that FN-1 has the highest number of nodes (Figure 8B). So, it could affect the growth of SF, which would have a considerable influence on the occurrence of RA.

## Discussion

RA is a long-term, widespread autoimmune condition, mainly impacting synovial joint membranes. It is also related to socioeconomic consequences, premature deaths, and increased impairment. However, the molecular mechanisms underlying the onset and progression of RA remain unknown. ScRNA-seq is a new method for sequencing the genome, transcriptome, and epigenome at the level of a single cell, enabling the detection of heterogeneous information from mixed samples that are not accessible by traditional sequencing (Hwang et al., 2018). In recent years, scRNA-seq has undergone significant advancements, and researchers now have the valuable opportunity to analyze the scRNA-seq datasets for sequenced samples from public repositories, thereby facilitating investigation of essential hub genes and signaling cascades from multidimensional perspectives (Ginhoux et al., 2022; Song et al., 2022). Herein, the gene analysis of SFs from RA patients was carried out using GSE109449 available in GEO. A total of 192 SFs from two RA patients’ scRNA-seq data were obtained using GEO, with cells implicated within such process examined using nFeature, nCount, and percent. mt. Additionally, the top 1,000 genes among the various cells with high expression aside from in mitochondria were asserted.

PCA is a popular technique for data analysis that uses a linear transformation to break down the original data into a set of linearly independent representations of each dimension. This technique can be used to isolate the data’s key feature components and is frequently employed to reduce the dimensionality of highly dimensional data (Zhou and Troyanskaya, 2021). In this study, TSNE analysis and PCA were both used for the top 1,000 genes. The important genes related to RA onset were further revealed by bioinformatic analysis, which was used to pinpoint the highly enriched signaling pathways connected to the marker genes. Three cell types (0,1, and 2) were identified by TSNE and some marker genes were found to be statistically upregulated in cell type 1 than the other cell types. These marker genes were highly enriched in the PI3K/AKT signaling cascade, which has previously been well-documented as being closely related to the functional activation of SFs, as well as in the organization of ECM, ECM containing collagen, and ECM structural components (Wang et al., 2018; Su et al., 2019).

Noticeably, FN-1 was verified as a crucial gene closely associated with the growth of SFs and has huge potential for regulation of RA initiation. FN-1, an ECM protein produced by fibroblasts, was previously found to be involved in the pathological changes in many tissues (Liu et al., 2019; Baik et al., 2022). Furthermore, emerging evidence has revealed that FN-1 is closely related to RA initiation and development (Przybysz et al., 2007; van Beers et al., 2012; Xu et al., 2021). In recently published studies, a systematic investigation of the functions, pathways, and networks of the genes related to RA was carried out. The findings showed that FN-1 is one of the hub genes in regulating the effectiveness of methotrexate treatment for RA patients (Wang et al., 2021). In the present study, FN-1 was identified as one of the most crucial genes related to RA initiation via a multiple analysis based on the scRNA-seq technique. Recently, there is a increasing number of evidence indicating that PI3K/AKT signaling affect the proliferation and oxidative stress of multiple cells. Interestingly, the role of this signaling on autoimmune inflammation in the synovial fibroblasts of RA is further reported (Hao et al., 2021). It was showed that antioxidant glutathione (GSH) could suppress the expressions of IL-6, IL-1β and other inflammatory factors, acting as an inflammatory suppressor by downregulating the secretion of reactive oxygen species and PI3K/AKT signaling in synovial fibroblasts. This study provides a new evidence for explaining the prominent role of PI3K/AKT signaling in mediation the development of RA in the future.

## Conclusion

Taken together, a multiple analysis was conducted upon scRNA-seq datasets for SFs in RA patients. The obtained results revealed that RA initiation-related genes were mainly enriched in ECM organization, collagen-harboring ECM, ECM structural constituent, and PI3K/AKT signaling cascade. In addition, FN-1 was identified as the key gene in the regulation of RA initiation, providing new light on OA pathophysiology and facilitating the identification of a possible therapeutic target for RA.

## Data Availability

Publicly available datasets were analyzed in this study. This data can be found here: https://www.ncbi.nlm.nih.gov/geo/query/acc.cgi?acc = GSE109449.
